# Metabolomics in autoimmune hepatitis: progress and perspectives

**DOI:** 10.3389/fmed.2025.1667391

**Published:** 2025-11-04

**Authors:** Xiaotong Huang, Wenhui Mo, Xiaoning Wang

**Affiliations:** ^1^Basic Medical College, Naval Medical University, Shanghai, China; ^2^Foshan Fosun Chancheng Hospital, Foshan, Guangdong, China; ^3^Institute of Interdisciplinary Science, Shanghai University of Traditional Chinese Medicine, Shanghai, China; ^4^Institute of Liver Disease, Shuguang Hospital, Shanghai University of Traditional Chinese Medicine, Shanghai, China

**Keywords:** autoimmune hepatitis, metabolomics, microbiome, artificial intelligence, diagnosis

## Abstract

This review summarizes recent advances in applying metabolomics to autoimmune hepatitis (AIH). AIH is a chronic liver disease characterized by immune-mediated hepatocellular injury, with complex pathogenesis involving genetic, immunological, and environmental factors. Metabolomics, a system-wide approach analyzing small molecule metabolites, offers potential in early diagnosis, prognosis, and therapeutic evaluation of AIH. Current studies identify alterations in amino acid, lipid, carbohydrate, and bile acid metabolism, as well as changes in the gut microbiome and specific metabolite markers that distinguish AIH from other liver diseases. Techniques such as liquid chromatography-mass spectrometry (LC–MS), and bioinformatics facilitate biomarker discovery and enhance understanding of disease mechanisms. Despite challenges such as standardization and data integration, metabolomics holds promise for developing personalized treatment strategies and advancing disease management. Future prospects include combining multi-omics approaches, large-scale cohort studies, and artificial intelligence (AI)-based data analysis to deepen insights into AIH pathology and improve clinical outcomes.

## 1 Introduction

Autoimmune hepatitis (AIH) is a chronic hepatocellular injury mediated by abnormal autoimmune responses. It is more common in women and is characterized by high gamma-globulinemia, positive serum autoantibodies, and a good response to immunosuppressive therapy (1). AIH was first identified by a Swedish physician in the 1950s (2). The disease can affect children and adults of all ages, but is most prevalent among individuals aged 40–70 years, particularly middle-aged women (3). The annual incidence of AIH is approximately 1–2 per 100,000 people, with a prevalence of 10–30 per 100,000 (4). In Europe, the prevalence ranges from 15 to 25 per 100,000 people, while in the Asia-Pacific region, it ranges from 4 to 24.5 per 100,000, with an annual incidence of 0.67–2.0 per 100,000 (5, 6). Currently, epidemiological data on AIH in China are lacking.

Clinically, AIH is divided into two subtypes based on the autoantibodies present: Type 1 AIH and Type 2 AIH (7). Type 1 AIH is associated with HLA-DR3 and HLA-DR4 and is characterized by positivity for antinuclear antibodies (ANA) and/or anti-smooth muscle antibodies (ASMA). It accounts for approximately 90% of cases, with peak onset around ages 10–18 and around 40 years. Type 2 AIH is associated with HLA-DR3 and is characterized by positivity for anti-liver−/kidney microsome 1 (anti-LKM-1) and/or anti-liver cytosol type 1 antibodies (anti-LC-1). It primarily affects adolescents and children, has an acute onset, a more severe course, and is prone to recurrence.

## 2 Pathogenesis of AIH

AIH is understood to arise from a confluence of genetic, epigenetic, and environmental factors. A well-documented genetic susceptibility is conferred by HLA alleles, particularly HLA-DR3 and HLA-DR4, with risk heterogeneity observed across different ethnic and regional populations. Beyond the genetic sequence, epigenetic mechanisms—evidenced by DNA hypo-methylation in CD4 + and CD19 + T cells and the association of microRNAs like miR-21 and miR-122 with inflammatory activity—further modulate disease expression. The genetic architecture is also shaped by single nucleotide polymorphisms within genes governing pro-inflammatory and regulatory pathways, such as tumor necrosis factor and CTLA-4/CD28. Exogenous triggers, including specific viral infections (e.g., hepatitis viruses, measles) and pharmacological agents (e.g., nitrofurantoin, minocycline), are thought to potentially initiate the autoimmune process through mechanisms like molecular mimicry, a role that has also been tentatively ascribed to vaccination. Notably, perturbations in the gut microbiome, characterized by an expansion of Veillonella and a reduction of Bifidobacterium, have been identified in AIH patients, positioning the microbiota as a contributor to pathogenesis and a potential target for therapeutic manipulation (8).

## 3 Clinical features and diagnosis

The diagnosis of AIH relies mainly on clinical manifestations, laboratory tests, and characteristic histopathological features of liver tissue. Two primary scoring systems are currently used for diagnosing autoimmune hepatitis (AIH): the revised original and the simplified criteria. The revised system demonstrates superior sensitivity (100% vs. 95%), whereas the simplified system offers better specificity (90% vs. 73%) and overall accuracy (92% vs. 82%). While the simplified system is valuable for excluding AIH in patients with other liver conditions, it must be applied with careful clinical judgment (9), as it may fail to identify atypical presentations (8, 10). Furthermore, both systems have inherent limitations: their diagnostic precision is compromised in acute or fulminant liver failure, and they inadequately account for atypical autoantibodies, reducing their efficacy in overlap syndromes. It is noteworthy that the Paris criteria demonstrate superior diagnostic accuracy compared to these AIH-specific scores. Additionally, a significant gap remains, as no clinical prognostic scoring systems for AIH are currently available (11).

### 3.1 Clinical manifestations

Most AIH patients have no obvious symptoms or only nonspecific symptoms such as fatigue. The disease often has an insidious onset, although a small number present with acute onset. Some patients experience acute exacerbation of chronic AIH, which may progress to acute liver failure. Approximately one-third of patients exhibit signs of cirrhosis at the initial diagnosis (12).

### 3.2 Laboratory examinations

Key laboratory features include elevated serum aminotransferase levels, positive autoantibodies, and increased serum immunoglobulin G (IgG) and/or gamma globulin levels (1).

#### 3.2.1 Serum biochemical indicators

Typical abnormalities involve hepatocellular injury, with elevated alanine aminotransferase (ALT) and aspartate aminotransferase (AST) levels. Alkaline phosphatase (ALP) and gamma-glutamyl transferase (GGT) are generally normal or slightly elevated. In severe or acute cases, serum total bilirubin (TBil) may be significantly increased.

#### 3.2.2 Autoantibodies

Most patients display high-titer autoantibodies, though these are often not disease-specific. Type 1 AIH typically exhibits positivity for ANA and/or ASMA, whereas Type 2 presents with anti-LKM-1 and/or anti-LC-1 antibodies (Table 1).

**Table 1:** Specificity and sensitivity of key AIH autoantibodies and overlap with other diseases.

Autoantibody	Specificity for AIH diagnosis	Sensitivity for AIH diagnosis	Overlap with other diseases
ANA	Limited overall due to similarity with other autoimmune diseases	Common in AIH-1; rare in AIH-2	Overlaps with multiple autoimmune diseases (e.g., SLE)
SMA	High with VG/VGT patterns	Variable in AIH patients	Seen in viral/extra-hepatic autoimmune diseases
Anti-LKM1	Specific for AIH-2	Present in some AIH-2 cases	Found in 10% of HCV patients
Anti-LKM3	Specific in AIH-2	In 10% of AIH-2 patients	Present in HDV
Anti-LC1	Can be the only marker for AIH-2	Often with anti-LKM1	Rare in HCV
Anti-SLA/LP	Highly specific (specificity: 99%)	In 15–30% of AIH-1; Rare in AIH-2	–
pANNA	Moderate in AIH-1	Common in AIH-1; Rare in AIH-2	More in PSC/IBD
Anti-ASGPR	Not specific	High in AIH patients	Also in viral/drug-induced hepatitis, PBC

Adapted from (48, 49).

#### 3.2.3 Serum immunoglobulins

Elevations in IgG and/or gamma globulins are characteristic of AIH.

### 3.3 Liver histology

Liver biopsy reveals hepatocellular injury, characterized by interface hepatitis, lymphoplasmacytic infiltration in portal and periportal areas, cellular infiltrates, and “rosette” formations of hepatocytes—hallmark features of AIH.

## 4 Pathogenesis and metabolic features of AIH

**Figure 1:**
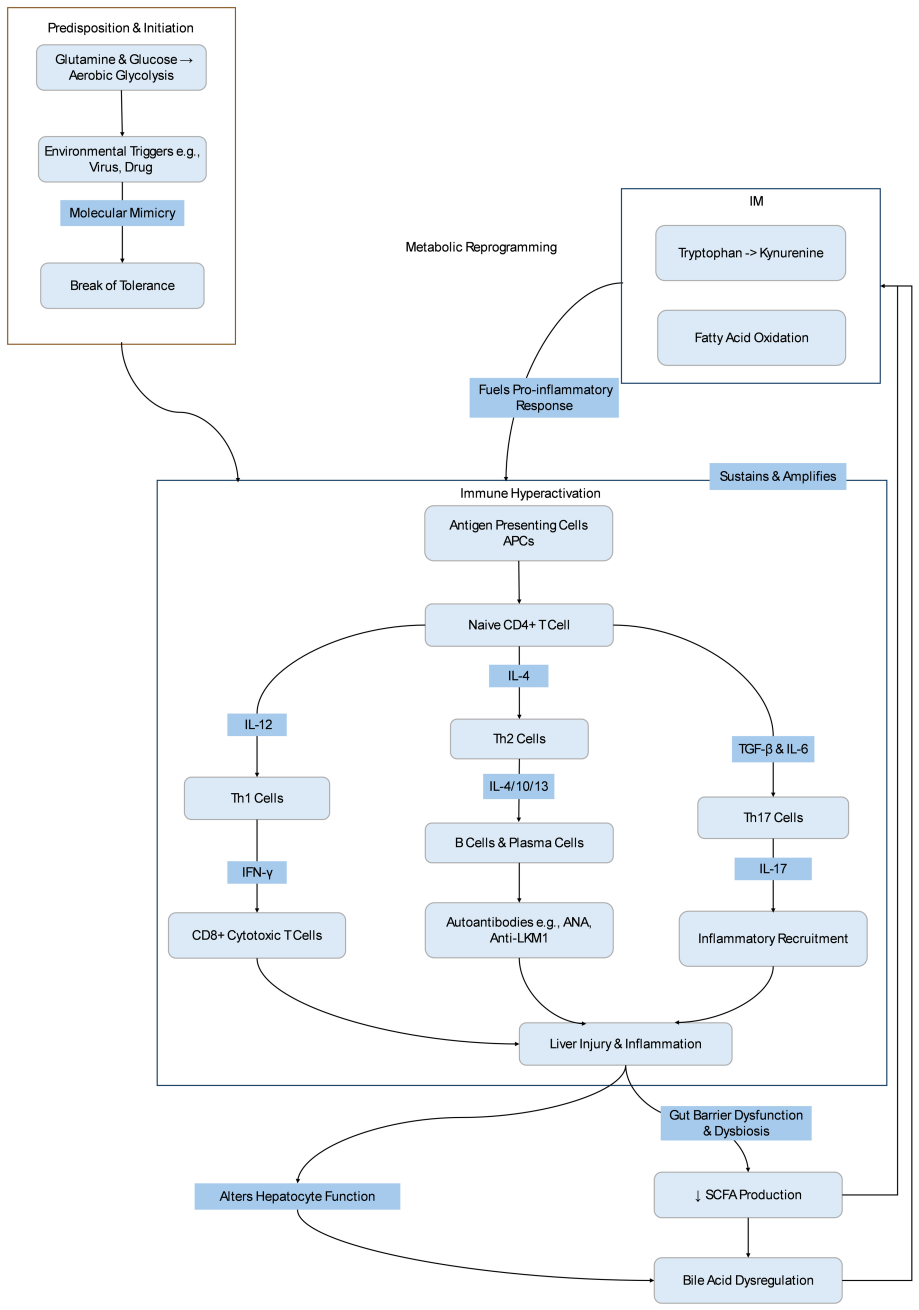
The self-sustaining cycle of immune-metabolic dysregulation in AIH. Adapted and revised from (1, 14).

The exact pathogenic mechanisms of AIH are not fully understood. Genetic, immunological, and environmental factors all play critical roles. These factors can induce T cell-mediated immune attacks against hepatic antigens, leading to AIH (Figure 1).

### 4.1 Molecular mimicry

In genetically susceptible individuals, immune responses against hepatic self-antigens may be triggered by molecular mimicry. Pathogens share structural similarities with self-proteins, causing immune reactions that mistakenly target self. Mouse models of Type 2 AIH have confirmed this mechanism; for example, mice stimulated with adeno-associated virus vectors expressing CYP2D6 produce anti-LKM-1 antibodies (13).

### 4.2 Self-antigen presentation and immune activation

AIH is initiated when self-antigens are presented to naïve CD4 + T cells (Th0). Antigen-presenting cells (APCs), such as dendritic cells (DCs), process self-antigens (AG) and present them to T cell receptors (TCRs) on initial Th0 cells, leading to the secretion of pro-inflammatory cytokines such as IL-4, IL-12, and TGF-*β*. In the presence of IL-12, Th0 cells differentiate into Th1 cells, which produce IL-2 and IFN-*γ*, activate cytotoxic CD8+ T cells, and induce IFN-γ and tumor necrosis factor-alpha (TNF-*α*). Exposure of hepatocytes to IFN-γ can upregulate MHC class I molecules and abnormally express MHC class II molecules, further activating T cells and causing ongoing liver damage. In the presence of IL-4, Th0 cells differentiate into Th2 cells, which secrete IL-4, IL-10, and IL-13. These cytokines promote B cell maturation into plasma cells that secrete autoantibodies. Antibody-dependent cellular cytotoxicity and complement activation contribute to tissue damage. When TGF-*β* dominates, Th0 cells differentiate into Th17 cells, which secrete IL-17. IL-17 induces inflammatory cytokines such as TNF-*α* and IL-6, promoting recruitment and infiltration of inflammatory cells, thus triggering immune-inflammatory responses and contributing to the development of AIH and hepatic fibrosis (1, 14, 15).

Further research emphasizes the role of immune cells, particularly CD4 + helper T cells (Th cells), in the response to AIH treatment. Patients with good treatment responses show increased proportions of anti-inflammatory Th2 cells and regulatory T cells (Tregs), while the proportions of pro-inflammatory Th1 and Th17 cells decrease. This finding underscores the importance of immune regulation in AIH therapy and may guide future immunotherapeutic strategies targeting these cell populations.

### 4.3 Immune tolerance imbalance

In healthy individuals, circulating autoreactive T cells exist, but intrinsic and extrinsic peripheral immune tolerance mechanisms prevent these cells from causing tissue damage. Tregs are crucial for inducing and maintaining immune tolerance. They suppress effector T lymphocyte proliferation and differentiation through direct contact and by secreting perforin and granzymes, exerting negative immune regulation. Studies suggest that an imbalance between Tregs and effector T cells (Teff) is critical for the occurrence and progression of tissue damage in AIH (16).

### 4.4 Metabolic features of AIH

The metabolic characteristics of AIH reflect the physiological and biochemical changes in the body in the disease state. Here are some known metabolic features of AIH:

#### 4.4.1 Amino acid metabolism

AIH patients often exhibit alterations in amino acid metabolism, especially the levels of certain essential amino acids may be elevated or decreased. This may be related to the reduced ability of the liver to synthesize proteins. The amino acid metabolic disturbances in AIH exhibit multidimensional characteristics, involving the dysregulation of key metabolic pathways and their dynamic interactions with immune responses. Disruption of tryptophan metabolism can compromise immune homeostasis: Gut microbiota break down tryptophan into indole derivatives (such as indole-3-aldehyde, I3A; kynurenine, KYN), which activate the aryl hydrocarbon receptor (AhR), driving CD8 T cell differentiation into IFNγ-secreting Tc1 cells, forming a positive feedback loop that exacerbates inflammation (17, 18). Methionine and its metabolite homocysteine also increase in the serum of AIH patients. Methionine metabolic disorders may induce inflammation and oxidative stress, while homocysteine can also trigger oxidative stress, promoting hepatitis progression and hepatocyte apoptosis (19).

Branched-Chain Amino Acid (BCAA) catabolism rate is increased in AIH (18, 20). One study showed that AIH patients have more aromatic amino acids and fewer BCAAs relative to healthy individuals (21). This mechanism may involve excessive activation of the mTOR and NF-κB pathways, increasing oxidative stress and inflammatory responses (22). Arginine and proline metabolism may be inhibited in AIH patients (20, 23). Arginine metabolic defects lead to reduced polyamine synthesis, which is unfavorable for the differentiation and maturation of intestinal resident immune cells, affecting intestinal immune regulation. At the same time, as a precursor of nitric oxide (NO), arginine metabolic imbalance will trigger an increase in nitric oxide (NO) synthesis, aggravating hepatocellular oxidative damage by inducing mitochondrial reactive oxygen species (ROS) production (24). In addition, abnormal proline metabolism leads to mitochondrial electron transport chain dysfunction through a proline dehydrogenase (PRODH)-dependent mechanism, triggering hepatocyte apoptosis (25).

Prior studies have found that L-glutamine levels may be elevated in AIH mice, and L-glutamine levels are positively correlated with indicators of liver injury (26). This may be because glutamine enters the tricarboxylic acid cycle (TCA cycle) by converting to *α*-ketoglutarate (α-KG), providing energy and biosynthetic precursors for T cells. Suppressing glutamine metabolism can reduce the differentiation and function of Th1 and Th17 cells, affecting T cell activation and differentiation, leading to AIH progression (27). The key intermediate product of purine nucleotide metabolism, inosine, is significantly lower in the livers of HLA-DRB1*04:05 positive patients than in negative patients. Inosine may affect the development of AIH by protecting hepatocytes and inhibiting over-activated immune cells (28). The progression of AIH causes the immune system to compete with other systems for energy, resulting in a large consumption of lipids and proteins. The increase of L-ketoalanine is related to inflammation and energy consumption. Glutaminylphenylalanine, hydroxyprolyl-tyrosine, and glutaminyltryptophan, and incomplete breakdown products of protein catabolism increase significantly in decompensated cirrhosis (29). These metabolic features provide potential targets for AIH diagnosis and intervention.

#### 4.4.2 Lipid metabolism

AIH patients often exhibit abnormal lipid profiles, including altered free fatty acids, triglycerides, and cholesterol levels, related to inflammatory processes, bile acid homeostasis disruption, and membrane damage.

Bile acid metabolism reprogramming is particularly significant in AIH. Serum levels of conjugated bile acids are significantly increased, including taurocholic acid and taurochenodeoxycholic acid. 7α-hydroxycholesterol (a catalytic product of Cyp7a1) is significantly reduced in patients with decompensated cirrhosis, suggesting decreased bile acid synthesis. Decreased bile acid synthesis may lead to dysregulation of enterhepatic circulation dynamics, further exacerbating liver inflammation.

Glycerophospholipid metabolism imbalance manifests as a significant decrease in serum levels of various lysophosphatidylcholines (lysoPCs) and lysophosphatidylethanolamines (lysoPEs). These glycerophospholipids are important components of hepatocellular and mitochondrial membranes. The decrease in their levels reflects the rapid turnover of hepatocellular membrane phospholipids, suggesting that cell membrane structure and function may be damaged during the early stages of liver injury.

Studies have also observed changes in serum levels in mice with liver injury induced by concanavalin A of certain long-chain acylcarnitines, and a significant reduction in a series of metabolites related to long-chain fatty acids, which may reflect changes in increased fatty acid oxidation and energy metabolism, or may be related to immune responses and energy consumption for immune cell activation and proliferation (29, 30). In addition, liver fatty acid binding protein (L-FABP), which is related to the metabolism of long-chain fatty acids, is an abundant small protein in hepatocytes that binds most long-chain fatty acids present in the cytoplasm. Previous studies have shown that these proteins are related to tissue damage. A recent prospective study showed that serum and/or urinary L-FABP levels and the good correlation between AST, ALT, CRE, and GGT can be used for the diagnosis of liver damage, including AIH (31).

#### 4.4.3 Carbohydrate and sugar metabolism

AIH patients may develop insulin resistance and glucose metabolism disorders, leading to fluctuations in blood glucose levels. Glucose metabolism abnormalities in AIH are manifested by the synergistic effect of immune cell metabolic reprogramming and hepatic parenchymal cell energy homeostasis imbalance, driving inflammation progression and liver injury. Gut microbiota-host interactions through metabolite regulate glucose metabolism. In AIH disease model and patients with acute hepatitis, a lack of short-chain fatty acids composed of propionic acid, acetic acid, and butyric acid may lead to intestinal mucosal barrier destruction (6, 32), inhibiting hepatocyte AMPK activation and weakening blood glucose level stability (33). On the other hand, these glucose metabolism abnormalities may reflect the energy competition between immune cells and hepatocytes. Damaged Tregs are considered a driving factor in AIH development. In AIH patients and mouse models, Tregs have enhanced glycolysis activity and weakened oxidative phosphorylation, resulting in impaired immunosuppressive function (34). Glycolysis is also enhanced in activated CD4 + T cells, promoting the release of inflammatory factors and liver damage (35). Compared with healthy volunteers and other liver disease patients, AIH patients have relatively higher levels of plasma pyruvate, lactate, acetate, and acetoacetate. These metabolic changes may demonstrate the aerobic glycolysis associated with the aforementioned excessive immune activation (21).

#### 4.4.4 Gut microbiome

The gut microbiome of AIH patients may change, which may affect the production and absorption of metabolites, thereby affecting the metabolic status of the liver. The gut microbiome dysbiosis in AIH is manifested by disordered flora structure, abnormal metabolic function, and imbalanced host-flora interaction, which jointly drive immune activation and liver damage. A significant reduction in flora diversity is a core feature: metagenomic analysis shows that the abundance of obligate anaerobic bacteria (such as Roseburia, *Faecalibacterium prausnitzii*) that produce SCFAs in the intestines of AIH patients is decreased, while potential pathogenic bacteria (such as Veillonella) are over-proliferated, and the abundance of *Veillonella dispar* is positively correlated with serum ALT levels and the degree of liver inflammation (23). Metabolic function remodeling includes: (1) Enrichment of tryptophan metabolic pathways, and (2) Enhanced lipopolysaccharide (LPS) biosynthesis. In a cross-sectional study of individuals with AIH (*n* = 91) and matched healthy controls (*n* = 98), lipopolysaccharide (LPS) is overexpressed in the AIH group relative to the control group (16), and the degree of elevation is significantly correlated with disease severity (23): Gut flora dysbiosis (such as increased abundance of Enterobacteriaceae) promotes LPS synthesis, stimulates immune responses, and induces liver injury (24). These findings highlight the core position of the gut microbiome as a “metabolic-immune regulator.” Future research needs to focus on strain-specific functions, fungi-virus community effects, and precise microbial intervention strategies.

#### 4.4.5 Others

Oxidative stress markers are usually elevated in AIH patients, indicating an imbalance of ROS and antioxidant substances. This state may aggravate liver damage and inflammatory response. Research shows that the levels of lipid and protein oxidation damage products are increased in AIH patients compared to the control group. Oxidizing components: levels of aldehydes, glutathione peroxidase activity, protein carbonyls, and isopropanol are significantly higher; and antioxidant components: whole blood glutathione levels gradually decrease with the progression from mild fibrosis to severe fibrosis and cirrhosis (36).

Additionally, some studies have identified specific metabolites, such as certain organic acids and steroid hormones, that have significant changes in AIH patients. These changes may be related to the severity and activity of the disease. Recent studies have found that AIH shows obvious high (sialylation per galactose on tetraantennary glycans, A4GS) galactose glycans on tetraantennary glycans sialylation ratio in plasma, which is a specific plasma N-glycan marker with potential impact on glycoprotein function and clearance rate (37). Reduced levels of retinyl ester in serum may be related to the activation of hepatic stellate cells (HSCs), which is one of the early signs of liver fibrosis, suggesting that disorders of retinal metabolism can be developed with liver fibrosis (30).

## 5 Overview of metabolomics

Metabolomics is a branch of systems biology that studies the comprehensive metabolite composition and changes of biological systems (such as cells, tissues or organisms) under specific physiological or pathological conditions. Metabolites are small molecule products in cellular metabolic processes, including amino acids, lipids, carbohydrates, nucleotides, vitamins, hormones and various intermediate metabolites. The goal of metabolomics is to provide a comprehensive chemical characterization of biological systems under different conditions, thereby revealing the metabolic basis of biological processes, disease mechanisms and drug effects. Common analytical techniques for metabolomics include nuclear magnetic resonance (NMR), gas chromatography–mass spectrometry (GC–MS) and liquid chromatography-mass spectrometry (LC–MS). Each method has its own specific application range and advantages. In practice, people also combine multiple technologies according to their needs to obtain to get more comprehensive metabolomics data. Metabolomics is widely used in early detection, therapeutic prediction and prognosis, monitoring treatment and recurrence detection of metabolic diseases. A large number of works have recorded the discovery of potential biomarkers and provided deeper insights into the pathogenesis of many human diseases.

### 5.1 Classification of metabolomics

Metabolomics is mainly divided into two modes: non-targeted and targeted. Non-targeted metabolomics has a wide detection range and requires combination with high-performance bioinformatics tools for analysis. It is mainly used to discover potential differential metabolic markers, but metabolite qualification is more difficult (38). Targeted metabolomics uses standards to quantitatively detect specific metabolites in samples, has high sensitivity and selectivity, and can be compared with known reference ranges to analyze the potential biological mechanisms of changes in differential metabolite (39).

### 5.2 Commonly used metabolomics analysis techniques and applications

Common analytical techniques for metabolomics include nuclear magnetic resonance spectroscopy (NMR), mass spectrometry (MS), gas chromatography (GC) and liquid chromatography (LC). In order to improve the sensitivity, resolution and selectivity of detection techniques, these techniques are often used in combination to optimize the workflow and provide potential for high-precision automated metabolomics analysis (40). Typical samples for metabolomics research are biological fluids, including blood, urine, sputum, cerebrospinal fluid, as well as feces, cells and various other tissues. Almost all diseases cause changes in metabolites, which makes quantitative metabolic profiling a practical method for discovering biomarkers.

### 5.3 Common data statistical methods for metabolomics

Discovering biomarkers is a basic application of metabolomics. In terms of data statistical analysis, univariate statistical analysis such as t-tests, and multivariate statistical analysis such as principal component analysis (PCA), partial least squares discriminant analysis (PLS-DA), orthogonal partial least squares discriminant analysis (OPLS-DA), etc. are currently used in metabolomics analysis. The main methods can effectively screen for disease diagnosis markers (41). Usually, we can use simple screening criteria such as fold change (FC), variable importance in projection (VIP), and *p*-values to select differentially expressed metabolites and analyze the differences between the study subject and the control, but strong correlations or commonalities are there are strong correlations or commonalities between these differentially expressed metabolites. Therefore, the use of more complex machine learning algorithms, such as LASSO, random forests (RF), especially those that can eliminate redundant and uninformative variables and reduce overfitting, to select key differential metabolites as potential biomarkers is also been widely used. In addition, univariate and multivariate logistic regression analysis of the screened markers, as well as further testing of the biomarkers and logistic models using independent validation cohorts, are of great significance for screening biomarkers.

## 6 Application of metabolomics in AIH research

Changes in metabolomic characteristics have also been reported in autoimmune diseases (Table 2). However, the metabolomic characteristics of AIH have not yet been fully elucidated, so we summarized the recent research progress on the metabolite characteristics of AIH patients.

**Table 2:** Candidate metabolic biomarkers in AIH research.

Biomarker category	Specific candidate metabolites	Reported changes in AIH	Potential clinical utility	Current validation status
Bile acids	Taurocholic acid, taurochenodeoxycholic acid	↑ Conjugated BAs	Early diagnosis; differentiation from PBC	Reported in multiple independent studies (29, 42); robust signal but needs standardization.
Glycerophospholipids	Lysophosphatidylcholines (lysoPCs)	↓ Multiple lysoPC species	Early diagnosis; indicator of hepatocellular membrane damage	Identified across different cohorts (29, 41); highly consistent finding.
Amino acids	Branched-chain amino acids (BCAAs)	↓ Relative to aromatic AAs	Disease diagnosis and monitoring	Reported in NMR and MS-based studies (17, 20); direction of change is consistent.
	Tryptophan metabolites (Kynurenine)	↑ Kynurenine pathway	Understanding immune-pathogenesis; Potential therapeutic target	Mechanistically linked to immune function (16, 17); more research needed for diagnostic use.
Energy metabolism	Pyruvate, lactate	↑ In plasma	Reflecting immune activation and aerobic glycolysis	Found in AIH vs. healthy controls (20); specificity against other liver diseases needs evaluation.
Microbiome-Linked	Short-chain fatty acids (SCFAs)	↓ Butyrate, propionate	Assessing gut-liver axis health; Potential therapeutic target (e.g., probiotics)	Indirect evidence from microbiome studies (6, 22); direct measurement in blood is challenging.
Treatment response	Azathioprine metabolites (6-TG, 6-MMP)	Variable (dose-dependent)	Clinically used for optimizing AZA dose (therapeutic drug monitoring)	Well-established and validated for utility in dose optimization (44, 45)

### 6.1 Diagnosis of AIH

Autoimmune hepatitis (AIH) is a complex liver disease, and the prevalence has been increasing in recent years. If patients are not accurately diagnosed or treated in a timely manner, they may develop severe or fulminant hepatitis. However, due to the lack of specific diagnostic markers and the significant heterogeneity of its clinical, laboratory and histological characteristics, accurate diagnosis of AIH, especially in the early stages, is still difficult. With the development of high-throughput methods, “omic” studies have become important to elucidate biological processes. As the products of cellular adjustment processes, metabolites levels are regarded as the ultimate readouts for genetic or environmental changes in biological systems (42). In one experiment, Metabonomic profiling was performed by ultra-performance liquid chromatography quadrupole time-of-flight mass spectrometry (UPLC Q-TOF MS). Fourteen metabolites were detected as potential biomarkers associated with early liver damage, including two bile acids, three long-chain acylcarnitines, seven glycerophospholipids, a bilirubin and a retinyl ester. Moreover, partial least square regression analysis showed that metabolism of glycerophospholipid, bile acids and retinol was highly correlated with the clinical outcomes, suggesting they played key roles in the early stage of the liver injury (30). In addition, analysis of N-glycosylation and IgG Fc glycosylation in plasma by mass spectrometry showed that specific glycosylation characteristics were significantly different in AIH patients from healthy controls (37).

AIH and primary biliary cholangitis (PBC) are both chronic cholestatic liver diseases caused by autoimmune system disorders. Because the clinical symptoms of the two are not typical, clinical differential diagnosis is relatively difficult. Therefore, recent AIH metabolomics research focuses on identifying biomarkers from human serum that can significantly distinguish between these two autoimmune liver diseases and healthy volunteers. These markers are mainly concentrated in several types of metabolites such as bile acids (BAs), free fatty acids, and phosphatidylcholines (42, 43). There have been studies using 1H-NMR to quantitatively analyze metabolites in the plasma samples of AIH patients, such as branched-chain amino acids, methionine, alanine-aspartic acid-glutamic acid and metabolites related to the intestinal flora (such as choline, betaine and dimethylamine), found there were significant differences between these metabolites in AIH and PBC patients (18).

Another experiment established a PBC and AIH differential diagnosis model using ultra-performance liquid chromatography-quadrupole time-of-flight mass spectrometry (UPLC-QTOF-MS), a new technology that can quickly and efficiently obtain detailed information about the properties of specific components in complex multi-component mixtures. Find out BAs levels can be used as markers to distinguish PBC and AIH, and these models may be useful for future research on the pathogenesis of PBC/AIH (43). More studies have compared the plasma metabolomic characteristics of patients, we provided proof-of-concept evidence that the 1H NMR analysis of plasma metabolites can be used to differentiate AIH patients from PBC, PBC/AIH overlap syndrome (OS), and drug-induced liver injury (DILI) overlap patients (21). Through with metabolomics, specific bacterial metabolic pathways and metabolites can be defined, and these findings may be important for the diagnosis and monitoring of AIH.

### 6.2 Prognosis and efficacy evaluation of AIH

Metabolomics is also widely used in the prognosis and efficacy evaluation of AIH: Yang et al.’s study revealed the complexity of treatment response in AIH patients through multi-omics methods including metabolomics, and identified lipid metabolites such as phosphatidylcholine (PC) as potential indicators for predicting treatment response (44).

Increasing evidence proves that monitoring the azathioprine (AZA) metabolites 6-thioguanine (6TG) and 6-methylmercaptopurine (6MMP) is beneficial for optimizing dosage. One study observed the metabolite profiles after combined use of allopurinol and low-dose thiopurine., illustrating that this combination method is an effective alternative for patients who have failed to achieve complete biochemical remission (CBR) (45, 46). Previous study demonstrated that there was an exactly therapeutic effect of Sancao granule (SCG), a traditional Chinese herb formula, on autoimmune hepatitis (AIH). Yang et al. By detecting serum biochemical indicators, it was found that SCG can regulate the expression of 9 metabolites related to 8 pathways, thereby exerting a good therapeutic effect on Con A-induced liver injury (47).

## 7 Challenges and prospects of metabolomics technology in AIH research

At present, the diagnosis of AIH is mainly based on clinical manifestations, laboratory tests and characteristic liver histology, and with the rapid development of metabolomics technology in recent years, these technologies have begun to be more widely used in the diagnosis and prognosis of AIH and efficacy assessment, becoming a research hotspot in academia.

The application of metabolomics in the diagnosis of AIH is constrained by several persistent limitations. Metabolomic data are profoundly influenced by pre-analytical and analytical conditions, including instrument parameters and sample processing procedures, resulting in substantial inter-laboratory variability. The imperative for stringent control over sample collection timing and storage conditions to preserve metabolite stability further restricts the reproducibility and accuracy of findings in clinical settings. Moreover, the current technological landscape lacks the capacity for the comprehensive detection of all metabolites. Compounding these issues is the limited specificity of candidate AIH biomarkers, which are frequently confounded by other hepatic disorders such as PBC and DILI, in addition to medications and other variables. These challenges collectively impede the development of a biomarker or a simple model capable of concurrently achieving high sensitivity and specificity.

The application of metabolomics technology in autoimmune hepatitis (AIH) research, although faced with many challenges, its potential and prospects are still promising. With the continuous advancement of technology, especially innovation in high-throughput analysis, data processing, and bioinformatics, metabolomics is expected to provide deeper insights for AIH research. First, standardized experimental methods and data analysis processes are key to improving the reproducibility and reliability of metabolomics research. Future research should be committed to establishing unified metabolomics analysis standards to facilitate comparisons and result verification between different studies. In addition, collaborative research will help integrate clinical data with metabolomics data, so as to more fully understand the pathological mechanism of AIH. Secondly, metabolomics has shown great potential in discovering new biomarkers. These biomarkers can be used not only for early diagnosis, but also to help assess disease activity and prognosis. Through large-scale cohort studies and longitudinal observations, researchers can identify specific metabolites associated with AIH, thereby promoting the development of personalized medicine. In addition, the application of metabolomics can also explore the relationship between AIH and other diseases, such as metabolic syndrome, diabetes, etc. This cross-disease analysis of metabolic characteristics helps to reveal common pathological mechanisms and provide new ideas and strategies for the treatment of multiple diseases. Finally, with the development of artificial intelligence and machine learning technologies, the analysis of metabolomics data will be more efficient and accurate. These technologies can help researchers extract valuable information from complex metabolic data, predict disease development, and provide support for clinical decisions.

In conclusion, despite existing challenges, metabolomics offers broad potential in AIH research. Ongoing technological innovations and multidisciplinary collaborations promise to unveil new insights—supporting early diagnosis, personalized therapy, and improved disease management. Combining metabolomics with other omics technologies will pave the way for comprehensive understanding and effective intervention strategies for AIH.
